# A Combination of Virulent and Non-Productive Phages Synergizes the Immune System against *Salmonella* Typhimurium Systemic Infection

**DOI:** 10.3390/ijms232112830

**Published:** 2022-10-24

**Authors:** Lu Liang, Jiaqi Huang, Ketong Cui, Peiyong Li, Wenjian Shi, Fang Lin, Guijuan Hao, Shuhong Sun

**Affiliations:** 1 Department of Preventive Veterinary Medicine, College of Veterinary Medicine, Shandong Agricultural University, Tai’an 271018, China; 2 Shandong Provincial Key Laboratory of Animal Biotechnology and Disease Control and Prevention, Shandong Agricultural University, Tai’an 271018, China

**Keywords:** *Salmonella* Typhimurium, phage cocktail, immunophage synergy, lysis from without, phage resistance

## Abstract

Effective phage cocktails consisting of multiple virus types are essential for successful phage therapy against pandrug-resistant pathogens, including *Salmonella enterica* serovar (*S*.) Typhimurium. Here we show that a *Salmonella* phage, F118P13, with non-productive infection and a lytic phage, PLL1, combined to inhibit pandrug-resistant *S.* Typhimurium growth and significantly limited resistance to phages in vitro. Further, intraperitoneal injection with this unique phage combination completely protected mice from *Salmonella*-induced death and inhibited bacterial proliferation rapidly in various organs. Furthermore, the phage combination treatment significantly attenuated the inflammatory response, restored the generation of CD4^+^ T cells repressed by *Salmonella*, and allowed macrophages and granulocytes to participate in immunophage synergy to promote bacterial clearance. Crucially, the non-productive phage F118P13 is less likely to be cleared by the immune system in vivo, thus providing an alternative to phage cocktail against bacterial infections.

## 1. Introduction

*Salmonella* has been recognized as a major and important foodborne pathogen for humans and animals for more than a century [1]. Following ingestion, *Salmonella* causes a multistage systemic infection ranging from gastroenteritis to invasive typhoid fever in both humans and animals [2]. Nontyphoid *Salmonella* spp. serovars, predominantly *Salmonella enterica* serovars (*S*.) Typhimurium and Enteritidis, primarily cause gastroenteritis, bacteremia, and focal infection, whereas typhoidal serovars (*S.* Typhi and *S.* Paratyphi A) mainly cause typhoid fever [3]. Among them, *S.* Typhimurium has been considered the prototypical broad-host-range serovar, it can cause acute inflammatory diarrhea that may progress to invasive systemic disease with bacteremia, meningitis, or focal infections that, if improperly treated, can be fatal [4,5]. Currently, non-typhoidal salmonella invasive disease most commonly occurs in infants and young children, older individuals, or immunocompromised or malnourished individuals, usually in sub-Saharan Africa, where the incidence of non-typhoidal salmonella invasive disease often exceeds 100 illnesses per 100,000 individuals per year [6,7]. In addition, multidrug resistant *Salmonella* isolates have been frequently identified from breeding farms, meat processing, and clinical trials in many countries [8,9,10,11,12], resulting in antibiotic treatment failure increasingly encountered for the emergence of pandrug-resistant isolates [13,14].

There has recently been a renewed interest in the use of bacteriophages (phages) for clinical and environmental applications. In contrast to antibiotics, phages are specific in their targets without directly affecting the normal microbiota of the host, and they are capable of auto-dosing for phage replication [15]. However, a major concern regarding the use of phages in the treatment of infectious diseases was the emergence of phage-resistant mutants [16,17]. When phage resistance develops in phage therapy using a single phage, this limitation can be overcome by using phage cocktails [18,19]. Previous attempts to use phage cocktails to prevent or treat bacterial infections mainly focused on broadening target bacterial strain spectrums, targeting multiple bacterial species, and limiting resistance to phages [20,21,22]. Katarzyna et al. [22] showed that a cocktail of two lytic phages given 1 h after *S.* Typhimurium challenge in a *Galleria mellonella* infection model increased the survival rate of larvae significantly more than a single-phage treatment. Studies by Bardina et al. [23] showed that a cocktail of three phages given 8 h before *S.* Typhimurium challenge or 4 h, 7 days, and 10 days after *S.* Typhimurium infection could not protect BALB/c mice from a systemic typhoid fever-like disease; the increased survival rate of mice was only observed when phages were administrated immediately after the challenge. Roach et al. [24] showed that host innate immunity was essential for the efficacy of phages in treating respiratory bacterial infections, defining the concept of “immunophage synergy”. Phage- and bacterial-derived pathogen-associated molecular patterns released after bacterial lysis have been proposed to stimulate local innate immune responses, which could promote bacterial clearance. These studies suggest that effective phage cocktail treatment of bacterial infections and the role of immune system in phage therapy merits further in-depth investigation.

In clinical practice, phages are typically administered as a mix of viral strains. However, pandrug-resistant bacteria are often faced with few available phages and poor therapeutic effects with screened phage isolates [12,25]. Furthermore, phages that exhibit abortive infection do not produce phage progeny, but the role of such phages in combating antibiotic resistant bacterial infections remains unclear. In the present study, we investigated a novel phage combination exhibiting both productive and abortive infection to control pandrug-resistant *S.* Typhimurium invasive systemic infections in a mouse model. Our results demonstrated that the phage combination successfully protected mice against *Salmonella* systemic infections with the help of the immune system, thus providing a novel alternative strategy of using a phage cocktail to treat non-typhoidal *Salmonella* invasive disease.

## 2. Results

### 2.1. Phages Effectively Inhibited Bacterial Growth In Vitro

**Figure 1:**
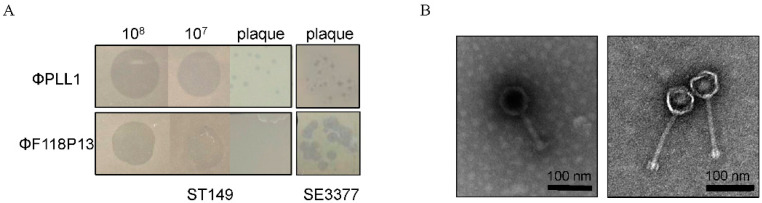
Morphological characteristics of ΦPLL1 and ΦF118P13. (**A**) Examples of phage plaque morphologies of ΦPLL1 and ΦF118P13 against different serotypes of *Salmonella*. (**B**) Transmission electron micrograph of two phages. ΦPLL1 (**left**), ΦF118P13 (**right**). Scale bar, 100 nm. Representative image is shown.

We screened 30 sewage samples to isolate virulent phages specific for pandrug-resistant *S.* Typhimurium strain ST149; however, only one phage, named PLL1, which can form small plaques on ST149, was obtained. Host range analysis showed that ΦPLL1 was specific for *Salmonella*, including *S*. Enteritidis, *S.* Typhimurium and *S*. Pullorum, whereas other members of *Enterobacteriaceae* that we tested cannot be lysed (Supplementary Table S1). In addition, ΦPLL1 exhibited similar EOPs on *S.* Typhimurium strain ST149 and *S*. Enteritidis stain SE3377 (Figure 1A). ΦF118P13, a virulent *Salmonella* phage isolated from the above sewage samples using *S.* Enteritidis as host, can form clear plaques on *S*. Enteritidis SE3377, but it is only able to produce clear zones over the ST149 lawn with high-titer (more than 10^7^ PFU/mL) droplets of phage particles, and no plaque was observed on *S.* Typhimurium using either phage titer (Figure 1A). Transmission electron microscopy (Figure 1B) revealed that ΦPLL1 possesses an icosahedral head, a neck without a collar, and a contractile tail, while ΦF118P13 has a capsid with a noncontractile tail with a length of ~120 nm. Genome sequence analysis confirmed that ΦPLL1 is a *Myoviridae* phage and ΦF118P13 is a *Siphoviridae* phage; both phages were virulent.

**Figure 2:**
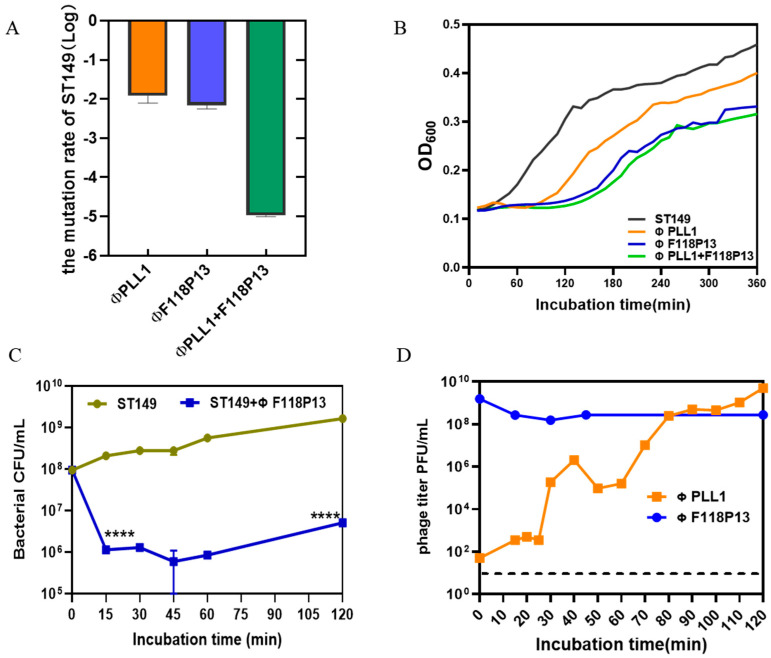
Comparison of the lytic activity of the phage combination and single phage alone on *S.* Typhimurium strain ST149 in vitro. (**A**) Mutation rates of ST149 resistance to phages. (**B**) Phages’ lytic activity on ST19 in vitro. Approximately 10^8^ *Salmonella* cells alone, or cells mixed with single phage or phage combination (MOI = 1) were incubated aerobically at 37 °C in 96-well plates. OD_600_ was measured at 10 min intervals for 6 h. (**C**) ΦF118P13’s lytic ability on ST149. Approximately 10^8^ *Salmonella* cells alone, or cells mixed with phages (MOI = 10) were incubated aerobically at 37 °C. Bacterial cells were determined by plating on LB plates. (**D**) The growth curve of ΦPLL1 and ΦF118P13’s proliferation in ST149. Approximately stationary phase 10^8^ CFU/mL of bacterial cells were incubated with ΦPLL1 and ΦF118P13 in LB medium at an MOI of 0.01 and 10, respectively. Phage titer was determined by plating on double-layer agar plates. **** *p* < 0.0001 (Student’s *t*-test).

To determine the rate of mutation of *S.* Typhimurium ST149 to ΦPLL1, ΦF118P13 and the combination of two phages, we used these phages to infect 10^8^ CFU of bacterial cells at an MOI of 1 for 2 h. Then the pelleted cells in the above cultures were diluted with phage-containing buffer for plating. The results showed that the *Salmonella* mutation rate to the phage combination reduced dramatically, to about 10^−5^: significantly lower than that of any single phage (both approximately 10^−2^) (Figure 2A). The bacterial growth curves showed that both ΦPLL1 and ΦF118P13 could effectively inhibit the growth of *S.* Typhimurium ST149 at MOIs of 1 and 10 (Supplementary Figure S1). However, the phage combination showed only a slight decrease in the optical density of bacterial cells compared to ΦF118P13 at an MOI of 1 (Figure 2B). The one-step growth curve of ΦPLL1 showed a latent period of approximately 15 min, and the burst size was 50 PFU per infected cell. Considering ΦF118P13 could only infect ST149 with high phage titer at MOIs of 1 and 10, we therefore tested the growth of ΦF118P13 at an MOI of 1. To our surprise, no progeny phage particles were produced during phage F118P13 infection; by way of contrast, the phage titer declined 10 times within 30 min at an MOI of 10, followed by bacterial cells with a reduction of 2 log_10_ CFU/mL (Figure 2C,D). These data suggest that these two phages had different ways to lyse *S.* Typhimurium: phage PLL1 lysed bacteria by lytic cycle with productive infection, whereas ΦF118P13 induced bacterial lysis with non-productive infection.

### 2.2. Phage Combination Protected Mice against Salmonella Systemic Infections

**Figure 3:**
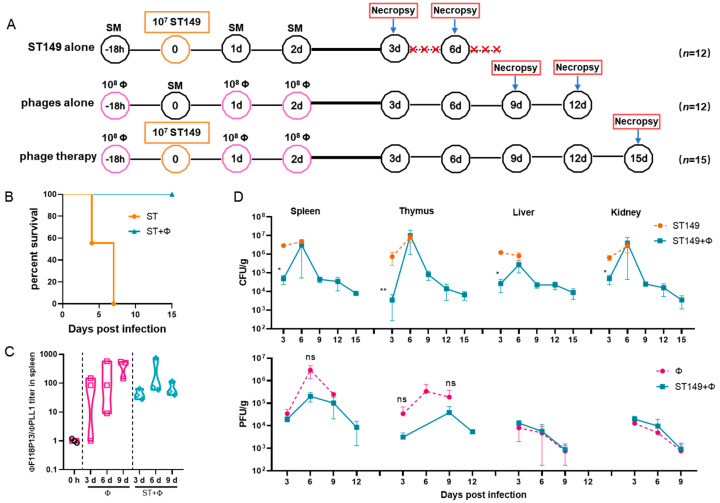
The phage combination protected mice against *Salmonella* systemic infections by inhibiting bacterial growth in organs. (**A**) Schematic depicting the experimental design, timeline, and treatment groups; × in red represents the number of dead mice. For the ST149 and phage combination control groups, 12 ICR mice were used in each group; for the phage combination-treated group, 15 mice were used. (**B**) Protection of the phage combination in infected mice. (**C**) The ratio of ΦF118P13 titer to ΦPLL1 titer in spleen in the phage alone group and the phage combination therapy group. (**D**) The bacterial load and phage titer in various organs. No data was obtained in the challenge group for 6 mice that died of *Salmonella* infection within 9 days. * *p* < 0.05 (Kruskal–Wallis test), ** *p* < 0.01; ns, no difference.

To establish a mouse model of *S.* Typhimurium invasive systemic infection, the challenge dose of pandrug-resistant strain ST149, which is lethal to ICR mice, was determined by intraperitoneal injection. It is worth noting that when *Salmonella*-infected mice were prostrate and unresponsive, preemptive euthanasia was performed and moribund animals would be euthanized by means of anesthetic isoflurane inhalation. The results showed that 10^5^ and 10^6^ of bacteria did not induce lethality in mice during the 14-day observation period. In contrast, the injection of 10^7^ cells was fatal in 16.7% of mice within 4 days and 83.3% by the end of 14-day of observation, and injection of 10^8^ cells quickly caused death in 100% of mice within 2 days (see Figure S2 in the Supplemental Material). Therefore, we used 10^7^ cells for challenge to examine whether the phage combination could protect against *S.* Typhimurium invasive systemic infection (Figure 3A). The result showed that phage therapy completely protected mice from *Salmonella*-induced death, while no mice survived in *Salmonella* challenge group at day 7 post-infection (Figure 3B). Figure 3C shows that the titer of ΦF118P13 in spleen was much higher than that of ΦPLL1 in both the phage alone group and the phage therapy group, implying that ΦF118P13 might play an important role in protecting mice from *Salmonella*-induced death. Mice treated with phages had a significantly reduced *Salmonella* burden in their spleen (4.7 log_10_ CFU/g), thymus (3.6 log_10_ CFU/g), liver (4.4 log_10_ CFU/g), and kidneys (4.7 log_10_ CFU/g), with a reduction of nearly 1.1 to 1.8 log at 3 days post-challenge relative to the levels (4.9~6.5 log_10_ CFU/g) in these organs of mice given *Salmonella* alone. By day 6, the numbers of bacteria had rebounded in all the above tested organs of mice treated with the phage combination, approaching the bacterial loads of corresponding organs in challenged mice without phage treatment. However, at the time of necropsy on day 9, the bacterial loads in organs of phage-treated mice showed a significant reduction, and kept decreasing to approximately 10^3^ CFU at day 15 (Figure 3D), while the phages’ titers gradually decreased to below the limit of detection in the liver and kidney from day 3 to day 12. Furthermore, the phage titers in the phage therapy group were notably lower than that in the phage control group.

**Figure 4:**
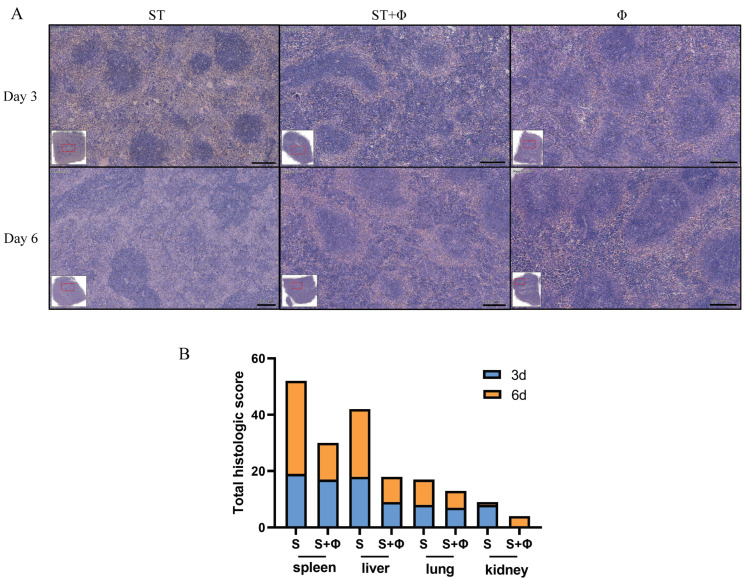
Phage combination alleviates tissue lesions. (**A**) Representative images of hematoxylin and eosin-stained spleen tissue from day 3 and day 6 post-infection. Bar = 200 μm. (**B**) Total histological scores of the spleen, liver, lung, and kidney harvested on day 3 and day 6 post-infection.

Histopathological examination showed massive hemolysis in the splenic red pulp, a reduction in white pulp area, and marked decreased lymphocytes in the spleen of *Salmonella*-challenged mice, whereas lymphocytes, neutrophils, and multinucleated giant cells in red pulp were markedly increased in mice treated with the phage combination (Figure 4A). Figure 4B shows that histological scores from organs of phage-treated mice were lower than those of infected mice that did not receive phage therapy, indicating that phage combination treatment prevented organ damage and reduced lesion severity.

### 2.3. The Immune System Contributed to Phage Therapy against Salmonella Infections

**Figure 5:**
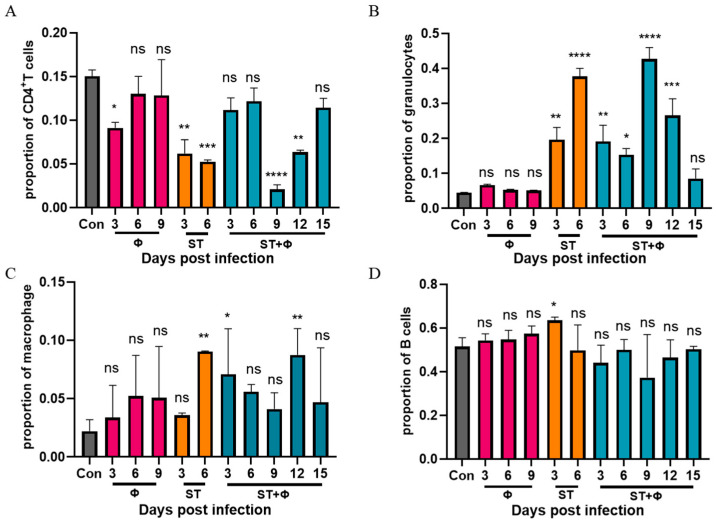
Phage therapy effect on immune responses in spleen in vivo. (**A**) Percentage of CD4^+^ T cells in live splenocytes. (**B**) Percentage of granulocytes in live splenocytes. (**C**) Percentage of macrophage in live splenocytes. (**D**) Percentage of B cells in live splenocytes. *, *p* < 0.05 (Kruskal–Wallis test), **, *p* < 0.01, ***, *p* < 0.001, ****, *p* < 0.0001; ns, no difference.

To explore why non-typhoidal salmonella invasive disease always occurs in immunocompromised individuals, we performed a flow cytometry approach to immunophenotype splenocytes of mice to understand the role of immune system in our successful treatment of *Salmonella* systemic infections. Since CD4^+^ T cells have been found to be important for the control of primary *Salmonella* infection and neutrophils are essential for the successful treatment of bacterial diseases with phages, we detected the percentage of CD4^+^ T cells and granulocytes in splenocytes. Figure 5A shows that *Salmonella* infection significantly reduced the percentage of CD4^+^ T cells by nearly three times within 6 days, whereas the percentage of CD4^+^ T cells underwent only a slight decrease in the phage therapy group. At day 9 post-infection, the number of CD4^+^ T cells decreased sharply to a very low level, then increased gradually over time and returned to approximate control levels at day 15 post-infection. For granulocytes (Figure 5B), a significant 3.4-fold and 7.4-fold increase in the number of granulocytes was observed in mice given *Salmonella* alone after 3 days’ and 6 days’ infection, respectively. In addition, the phage combination treatment retarded the relatively high increase of granulocytes caused by *Salmonella* infection in the first 6 days. However, the percentage of granulocytes rose sharply (8.5-fold) on the following day 9, then gradually returned to the control level at day 15 post-infection. Figure 5C shows that the percentage of macrophages in challenged mice increased continuously and peaked at day 6, while the proportion of macrophages, which also increased in the phage-treated group, reached the highest level at day 12. For B cells, as indicated in Figure 5D, a slight increase in infected mice at 3 days post-infection was found, but there was no significant difference in other groups. It is noteworthy that mice which received phages only showed no significant difference in CD4^+^ T cells, granulocytes, or B cells compared to the control group, but phages alone caused an increase in macrophages, indicating the uptake of phages into macrophages and subsequent macrophage activation.

### 2.4. Phage Combination Attenuated Salmonella-Induced Inflammatory Response

**Figure 6:**
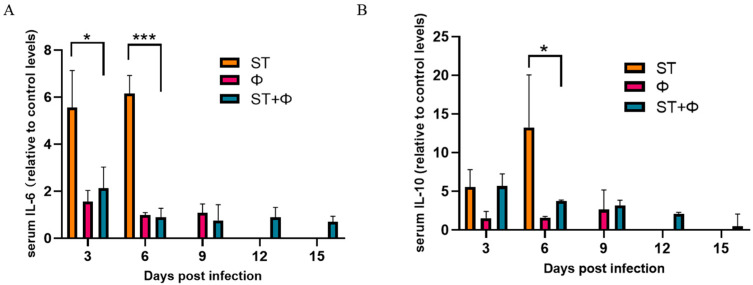
Phage therapy effect on inflammatory responses. (**A**) Serum levels of IL-6 on day 3 and day 6 post-infection relative to the level of IL-6 in the control group. (**B**) Serum levels of IL-10 on day 3 and day 6 post-infection relative to the level of IL-10 in the control group. *, *p* < 0.05 (ordinary one-way ANOVA), ***, *p* < 0.001.

We then investigated the host inflammatory response in serum during phage combination treatment. As indicated in Figure 6A, the serum level of IL-6 (a pro-inflammatory factor) in mice given *Salmonella* alone stayed at a high level within 6 days, whereas phage therapy significantly reduced the level of IL-6. IL-10, an anti-inflammatory cytokine, showed a gradual increase in mice given *Salmonella* alone and reached the highest level on day 6 post-infection. Although phage combination treatment showed a 3.6~5.0-fold increase in the level of IL-10 within 6 days, there was a relative 3.6-fold decrease at day 6 compared with infected mice (Figure 6B). The results indicated that phage combination may help to attenuate *Salmonella*-induced inflammatory response.

## 3. Discussion

Several studies have proposed multiple techniques to improve phage cocktails [18,26,27]. Here, we have shown that intraperitoneal administration of a novel *Salmonella*-specific phage combination protects against *Salmonella* invasive systemic infections by inhibiting the rapid proliferation and spread of bacteria in various organs and attenuating the inflammatory response in a mouse model, and that the elimination of *Salmonella* also depends on the host’s immune system. We have shown that ΦF118P13-induced bacterial lysis via high multiplicities adsorption with non-productive infection can be used to reduce phage-resistance mutations and kill *Salmonella* in vitro. The effect of phage combination lysis on *S.* Typhimurium synergizes the immune system to resolve acute invasive *Salmonella* systemic infections. Crucially, the phage with non-productive infection is less likely to be cleared by the immune system, supporting the novel strategy of a phage cocktail rather than the prevailing paradigms [18].

The development of resistance and the need for readily available phages are the two major hurdles with phage therapy [20,28]. Although the isolated ΦPLL1 in this study was able to significantly inhibit the growth of *S.* Typhimurium at low MOIs, the high rate of bacterial mutation to phage resistance still greatly increased the risk of single phage therapy, which may also be likely to occur in other clinical bacteria isolates with high mutation rates. For example, *Pseudomonas aeruginosa* often leads to insufficient efficacy of phage therapy due to its enormous capacity to engender resistance [29,30]. A phage that cannot form plaques on its host bacteria is usually not considered for phage therapy even if phage lysis occurs, and this kind of phage could be missed using the conventional phage isolation method. Here, we screened our phage libraries and obtained a lytic phage inducing *S.* Typhimurium lysis via lysis from without with non-productive progeny phage production, offering the possibility of this kind of phage being useful in treating acute *Salmonella* invasive systemic infections. The fact that the titers of ΦF118P13 were higher than those of virulent ΦPLL1 implies that the predominant phage protecting mice from *Salmonella*-induced death appears to be non-productive ΦF118P13, which can survive for more than 12 days in vivo, whereas virulent ΦPLL1 seemed more likely to be cleared by the immune system. The reason for the rapid decline of ΦPLL1 in vivo still requires further research; however, the potential of phages that exhibit abortive infections, to the best of our knowledge, in the treatment of bacterial infections has not yet been explored. While the mechanism of lysis from without is still unclear, our data implies that such phages’ ability to cause abortive infection might also offer an alternative to other phages or antibiotics in combating some intractable infections.

The initial stages of *Salmonella* infection are characterized by effective recruitment and activation of phagocytes, partly due to a massive inflammatory response in infected tissues, including the expression of inflammatory cytokines [e.g., IL-6, IL-12, and IL-18]. In addition, bacterial clearance is preceded by activation of T cells including CD4^+^ and CD8^+^ T cells [31]. Consistent with those of other studies, our findings show that ICR mice have intermediate susceptibility to infection with *S.* Typhimurium [32]. We therefore selected ICR mice instead of the more susceptible C57BL/6 mice, which lack the macrophage-encoded Nramp1 gene, to better study the role of the immune system. Invasive *Salmonella* systemic infections and organ damage lead to an uncontrolled inflammatory response and overactivation of neutrophils, and massive *Salmonella* further proliferates within macrophages and induces cell death, thus successfully adapting to the pressure imposed by the innate immune system. We have shown that *S.* Typhimurium restrains activation of CD4^+^ T cells in the acquired immune response, thus causing an overwhelming invasive systemic infection that leads to massive deleterious tissue injury culminating in the death of the host, although the initiation of host-adaptive B cell responses was found in infected mice. Alaniz et al. [33] demonstrated that *Salmonella* transitioning from an extracellular phase to an intracellular phase could reduce the ability of splenic antigen-presenting cells to present FliC to CD4^+^T cells, and thus escape detection by host adaptive immunity by restricting FliC bioavailability via antigen compartmentalization. Interestingly, phage combination treatment inhibited the rapid proliferation and spread of *S.* Typhimurium in various organs, thus preventing cytokine release syndrome and allowing immunophage synergy to clear bacteria. Recent studies have suggested that phages alone are unable to exterminate the whole bacterial population and that cooperation with the immune system is a prerequisite for successful phage therapy [24,34]. Although phages for the mouse model in this study were only filtered and not purified, remnant *Salmonella* debris in phage lysates induced the immune system to a certain extent; our results showed that when *Salmonella* continued to proliferate at 6 days post-infection, CD4^+^ T cells, macrophages, and granulocytes all participated to promote the reduction of bacterial load together in response to phage therapy. However, adaptive humoral responses (B cells) seemed not to play a great role in immunophage synergy in resolving *Salmonella* systemic infections. Another thing worth noting is that although great importance was attached to animal welfare and animal protocols were strictly followed in the mice model in this study, less drastic and ethically acceptable treatment should be used in the future to explore ways to effectively control bacterial invasive diseases. Thus, this animal model should not be replicated 1:1.

## 4. Materials and Methods

### 4.1. Bacterial Strains

*Salmonella* Enteritidis SE3377 was purchased from the China Veterinary Culture Collection Center (Beijing, China). Pandrug-resistant *S.* Typhimurium ST149, isolated from chickens in Guangdong Province of China, exhibited resistance to many antibiotics in penicillins, aminoglycosides, folate pathway antagonists, tetracyclines, and phenicols. Other *Salmonella* serotypes and other species of *Enterobacteriaceae* used in this work are described in the Supplemental material Table S1. All strains were cultured in Luria-Bertani (LB) medium or on LB agar plates at 37 °C, unless otherwise noted.

### 4.2. Phage Isolation, Propagation, and Characterization

The sewage samples used for phage isolation were collected from chicken farms, urban rivers, schools, restaurants, and shopping malls in Shandong Province. For phages isolation, the pandrug-resistant *S.* Typhimurium ST149 was used by standard enrichment techniques and double-agar overlay plaque assays described by Kropinski [35]. To preserve the phages, plaques were picked and dispersed into SM buffer (10 mM MgSO_4_, 100 mM NaCl, 0.01% gelatin, 50 mM Tris-HCl, pH 7.5) and stored at 4 °C. Meanwhile, other phages in our laboratory were screened by spot tests and double-layer agar methods to determine their lytic activity on ST149. The phage combination was prepared by mixing two phages at a ratio of 1:1, with each phage at a titer of 9 log10 PFU/mL, later diluted in sterile SM buffer to reach the target concentration for subsequent analysis. For measuring lysis activity of the phages against *Salmonella* strains, stationary cultures (10^8^ CFU/mL) were mixed with phages or phage combination at different MOIs (multiplicities of infection) in 96-well plates and incubated at 37 °C in a standing condition. Growth curves were monitored by measuring the optical density at 600 nm (OD_600_) at 10-min intervals for 6 h. The one-step growth curve of a phage and the frequency of phage-resistant mutants for strain ST149 were measured as described previously, with a slight modification [36,37]. Briefly, to determine the rate of mutation of *S.* Typhimurium ST149 to phages, approximately 10^8^ CFU of overnight bacterial cells were infected with phages at an MOI of 1 at 37 °C, 180 rpm for 2 h, then the cultures were centrifuged and the pelleted cells were resuspended with SM buffer containing 10^8^ PFU of corresponding phages. Serial dilutions containing phages were plated directly onto LB agar plates to count the number of phage-resistant mutants.

### 4.3. Transmission Electron Microscopy

High-titer phage lysates were prepared in LB medium based on the best MOIs of phages. The samples were then deposited on carbon-coated Formvar films and stained with 2% uranyl acetate. Microscopy was performed with a H7650 transmission electron microscope (TEM) (Hitachi, Tokyo, Japan).

### 4.4. Phage Genome Sequencing and Analysis

Extraction and purification of genomic DNA from *Salmonella* phage suspensions were carried out using a lambda phage genomic DNA Kit (Zoman Biotek Corp., Beijing, China), according to the manufacturer’s protocol. The whole-genome sequencing using Illumina NovaSeq PE150 was performed at Beijing Novogene Bioinformatics Technology Co., Ltd. (Beijing, China). Open reading frames (ORFs) were predicted using GeneMarkS and annotated for specific functions using the RAST and PHASTER programs [38,39]. BLASTP and PSI-BLAST searches were used to determine the similarity of all putative proteins in the genome sequence.

### 4.5. Salmonella Infection of Mice

All animal work was reviewed and approved by the Laboratory Animal Care Committee of Shandong Agricultural University [permit number SDAUA-2021-034]. However, this animal model should not be replicated 1:1 in regard to the animal welfare and the human endpoints. Male ICR mice (age, 5 weeks old) were purchased from Jinan Pengyue Experimental Animal Breeding Co., LTD (Jinan, China) for use in infection experiments. The food, bedding, and water were autoclaved. To determine the lethal dose for *Salmonella* infection in mice, *S.* Typhimurium ST149 was used for intraperitoneal injection with different doses in an inoculum of 100 μL (10^5^–10^8^ CFU) in six mice each. Once infected mice were prostrate and unresponsive, moribund animals were euthanized preemptively by means of anesthetic isoflurane inhalation. Dosed mice were observed for 14 days to report the mortality in each group.

The phage combination for the animal experiment was prepared by mixing two phages with a ratio of 1:1 in SM buffer, each phage at a titer of 9 log10 PFU/mL. To examine the therapeutic effect of phage combination on *Salmonella* infection, mice in the phage treatment group were injected intraperitoneally with 100 μL of above phages 18 h prior to the bacterial challenge, then phages were administrated at 24 and 48 h post-infection. For mice in *Salmonella* the challenge group, the phage combination alone group, and the phage treatment group were all monitored for 15 days, and three live mice in each group were euthanized with anesthetic isoflurane inhalation every three days until the endpoints. Tissue samples from spleen, liver, kidney, and lung were removed aseptically, weighed, homogenized in sterile PBS, diluted, and subjected to bacteriological examination (CFU count) on xylose lysine desoxycholate (XLD) agar plates. To determine the titer of total phage and ΦPLL1 in these organs, supernatants were filtered through filters with a pore size of 0.22 μm and serial dilutions were plated on double-layer agar containing *S.* Enteritidis SE3377 and containing *S.* Typhimurium ST149, respectively. The spleen was also harvested for histopathology and flow cytometry analysis. Blood was collected from the abdominal vena cava of mice and serum was used to detect the cytokines.

### 4.6. Histopathological Analysis and Scoring

At the time of necropsy, the spleen, liver, kidneys, and lungs were stored in 10% buffered formalin for 48 h and then transferred to 70% ethyl alcohol for long-term storage. Hematoxylin- and eosin-stained slides were prepared for histopathological examination using TissueGnostics TissueFAXS system (Vienna, Austria). Histological sections were coded, randomized, and scored based on a previously published numerical scoring scheme [40]. Lesion severity in the organs was scored on a scale of 0 (no obvious abnormality) to 3, and each organ’s features are described in Supplementary Table S2. These changes were also quantified in terms of the percentage involvement by the disease process: (0) no obvious abnormity, (1) 1–5%, (2) 6–40%, (3) 41–80%, and (4) 81–100%. The total histological score was scored for each feature separately by establishing the product of the grade for that feature and the percentage involvement.

### 4.7. Flow Cytometric Analysis

After splenocyte recovery treatment, aliquots of 10^6^~10^8^ cells/mL assessed using Trypan Blue were stained with saturating amounts of CD3-FITC (clone 17A2), CD4-eFlour 450 (clone GK1.5), CD19-PerCP-Cyanine5.5 (clone eBio1D3), and CD11b-FITC (clone M1/70) antibodies (Thermo Fisher Scientific, Waltham, MA, USA) for 30 min at 4 °C, according to the manufacturer’s instructions. The eBioscience™ Fixable Viability Dye was used for staining dead cells. After permeabilization, samples were washed twice, resuspended with PBS and immediately analyzed on an LSRFortessa Flow Cytometer (BD Biosciences, Franklin Lakes, NJ, USA). The flow data were processed using FlowJo software (Tree Star, Inc., Ashland, OR, USA).

### 4.8. Serum Cytokine Assays

Serum cytokines containing interleukin (IL)-6 and IL-10 were detected by enzyme-linked immunosorbent assay (ELISA) using ExCell Bio kits (Taicang, China) according to the manufacturer’s instructions.

## 5. Conclusions

To conclude, these data suggest that the phage combination (i) is efficient at killing *S.* Typhimurium cells in vitro and in vivo, (ii) prevents cytokine release syndrome, and (iii) provides protection against *Salmonella* systemic infection in the mouse model due to immunophage synergy. Application of the phage with non-productive infection provides a novel alternative strategy of phage cocktail in combating pandrug-resistant bacterial infections. Furthermore, our results also provide an insight in the tripartite interactions among phages, bacteria, and the host’s immune system.

## Data Availability

The complete genome sequences of ΦPLL1 and ΦF118P13 were deposited in the NCBI database under the GenBank accession numbers OM367911 and OM339548, respectively.

## Supplementary Materials

The supporting information can be downloaded at: https://www.mdpi.com/article/10.3390/ijms232112830/s1.
