# Supplementary material for: A Combination of Virulent and Non-Productive Phages Synergizes the Immune System against Salmonella Typhimurium Systemic Infection
Source: Int J Mol Sci. 2022 Oct 24;23(21):12830. doi: 10.3390/ijms232112830 (PMC9654417; doi:10.3390/ijms232112830)
Supplement: Supplementary file 1 [file ijms-23-12830-s001.zip › ijms-1975775-supplementary.pdf]

A

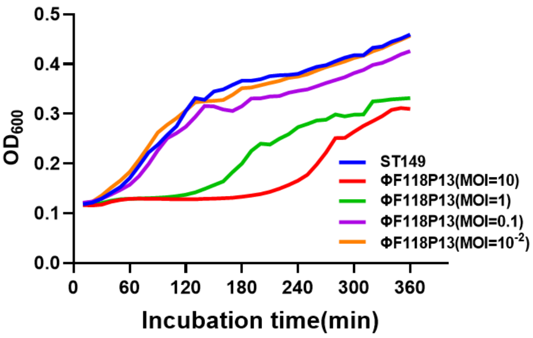

B

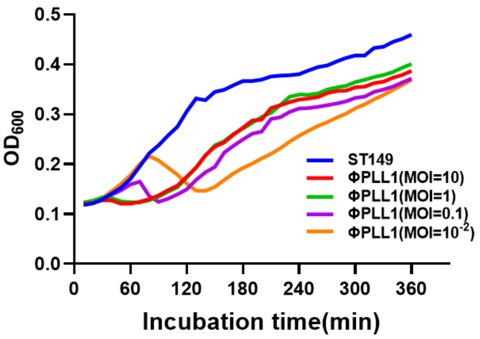

Supplementary Figure S1. Lytic activity of ΦF118P13 **(A)** and ΦPLL1 **(B)** on *S.* Typhimurium ST149 at different MOIs *in vitro*.

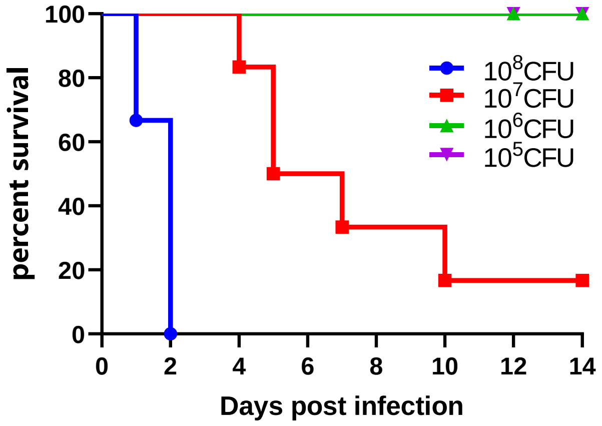

Supplementary Figure S2. The lethality of ICR mice by intraperitoneal injection with different doses of *S*. Typhimurium ST149.

Supplementary Table S1. Host range of *Salmonella* phage PLL1 and F118P13.

Strain	Bacteria species	Sensitivity to ΦPLL1[a]	Sensitivity to ΦF118P13
SPG8	*Salmonella* Pullorum	++	++
SPL4K	*Salmonella* Pullorum	-	-
ST722	*Salmonella* Typhimurium	++	++
ST149	*Salmonella* Typhimurium	++	++
ST699	*Salmonella* Typhimurium	++	++
ST18Y15	*Salmonella* Typhimurium	++	++
CVCC3377	*Salmonella* Enteritidis	++	++
SK18Y23	*Salmonella* Kentucky	-	-
SK18Y24	*Salmonella* Kentucky	-	-
SK147	*Salmonella* Kentucky	-	-
SL151	*Salmonella* London	-	-
JAF2	*Klebsiella pneumoniae*	-	-
JA4	*Klebsiella pneumoniae*	-	-
JA3-1	*Escherichia coli*	-	-
JAF1	*Escherichia coli*	-	-
FC2-2	*Proteus mirabilis*	-	-
T102-2	*Proteus mirabilis*	-	-

a: “++”, clear plaque, “-” no plaque.

Supplementary Table S2. Pathological feature scores used in this study.

Organ	Lesion	Score
Kidney	hyperplasia	1
	hemorrhage	2
	necrosis	3
Lung	hyperplasia	1
	exudation	2
	hemorrhage	3
Liver	organizational barriers	1
	cellular swelling	1
	inflammatory hyperplasia	2
	necrosis	3
Spleen	multinucleated giant cell hyperplasia	1
	exudation	2
	lymphocytosis	2
	white pulp reduction	3
	marginal zone reduction	3
